# The Bcl-2 Family: Ancient Origins, Conserved Structures, and Divergent Mechanisms

**DOI:** 10.3390/biom10010128

**Published:** 2020-01-12

**Authors:** Suresh Banjara, Chathura D. Suraweera, Mark G. Hinds, Marc Kvansakul

**Affiliations:** 1 Department of Biochemistry & Genetics, La Trobe Institute for Molecular Science, La Trobe University, Melbourne, VIC 3086, Australia; 17843636@students.latrobe.edu.au (S.B.); C.Suraweera@latrobe.edu.au (C.D.S.); 2 Bio21 Molecular Science and Biotechnology Institute, The University of Melbourne, Parkville, VIC 3052, Australia

**Keywords:** apoptosis, Bcl-2, evolution, mechanism, structure analysis

## Abstract

Intrinsic apoptosis, the response to intracellular cell death stimuli, is regulated by the interplay of the B-cell lymphoma 2 (Bcl-2) family and their membrane interactions. Bcl-2 proteins mediate a number of processes including development, homeostasis, autophagy, and innate and adaptive immune responses and their dysregulation underpins a host of diseases including cancer. The Bcl-2 family is characterized by the presence of conserved sequence motifs called Bcl-2 homology motifs, as well as a transmembrane region, which form the interaction sites and intracellular location mechanism, respectively. Bcl-2 proteins have been recognized in the earliest metazoans including Porifera (sponges), Placozoans, and Cnidarians (e.g., Hydra). A number of viruses have gained Bcl-2 homologs and subvert innate immunity and cellular apoptosis for their replication, but they frequently have very different sequences to their host Bcl-2 analogs. Though most mechanisms of apoptosis initiation converge on activation of caspases that destroy the cell from within, the numerous gene insertions, deletions, and duplications during evolution have led to a divergence in mechanisms of intrinsic apoptosis. Currently, the action of the Bcl-2 family is best understood in vertebrates and nematodes but new insights are emerging from evolutionarily earlier organisms. This review focuses on the mechanisms underpinning the activity of Bcl-2 proteins including their structures and interactions, and how they have changed over the course of evolution.

## 1. Introduction

**Figure 1:**
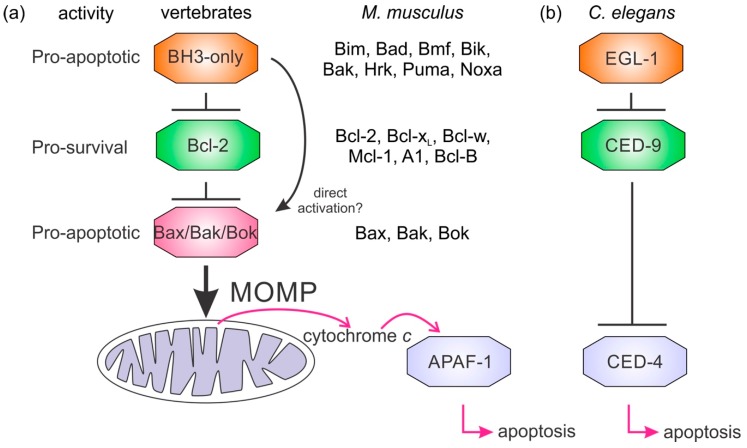
Mechanisms of Bcl-2 regulated apoptosis. Simplified apoptosis schemes showing the role of Bcl-2 proteins in apoptosis initiation differ between mammals (**a**) and nematodes (**b**). The Bcl-2 family members are indicated for *M. musculus.* Nematodes have a simplified activation of apoptosis where the BH3-only protein EGL–1 binds the sole prosurvival protein in the *C. elegans* genome CED-9. This event releases the caspase activating protein CED–4 to initiate the caspase cascade. In mammalian apoptosis, the BH3-only group of proteins antagonize the prosurvival Bcl-2 proteins releasing Bax, Bak, or Bok to oligomerize and form pores in mitochondria causing mitochondrial outer membrane permeabilization (MOMP). Cytochrome *c* release from the mitochondrion triggers the mammalian equivalent of CED-4, APAF-1, to oligomerize and initiate the activation of downstream caspases.

Apoptosis or programmed death of cells has played a significant role in metazoan evolution and prioritizes the organism over individual cells [1,2]. One form of apoptosis, intrinsic or mitochondrial regulated apoptosis, is initiated by a range of intra- and extracellular stimuli to regulate developmental and homeostatic processes [3]. The genes most closely associated with intrinsic apoptosis are the B-cell lymphoma 2 (Bcl-2) family and have been identified in the basal clades of metazoans, including Porifera (sponges), Cnidaria (anemones, corals, jellyfish), and Placozoa [4]. In mammals, these genes regulate the integrity of mitochondria where they either initiate the release of apoptogenic factors or prevent this process from occurring (Figure 1). The threshold for cell fate is mediated by antagonism between prosurvival and proapoptotic members of the Bcl-2 family [5] and this fundamental interaction is conserved from sponges [6] to man [7]. Evolutionary gene losses have led to simplification of this process in some organisms, such as insects and nematodes (Figure 1b). Viruses have also acquired Bcl-2 genes to facilitate replication and counter infected cells’ ability to orchestrate intrinsic apoptosis as part of antiviral defense mechanisms [8]. The molecular basis of the interaction between prosurvival and proapoptotic proteins relies on their conserved sequences and structures [7].

**Figure 2:**
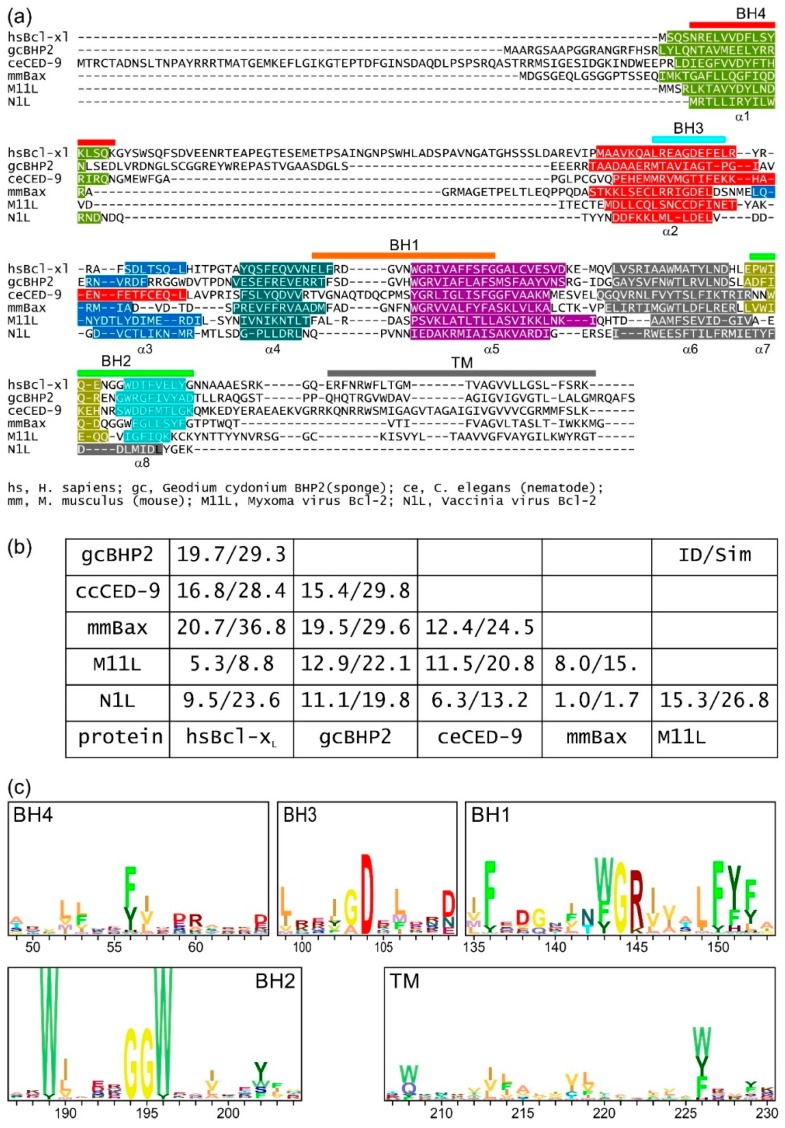
Sequence-structure analysis of Bcl-2 family members from sponges to man. (**a**) Structure-based sequence alignment of metazoan and viral Bcl-2 family members. Structurally equivalent residues are aligned. In (**a**), sequence–structure alignment shows prosurvival and proapoptotic Bcl-2 proteins share key sequence features. Sequences aligned: *H. sapiens* Bcl-x_L_; *G. cydonium* BHP2; *C. elegans* CED-9; *Mus musculus* Bax; Myxoma virus M11L; Vaccinia virus N1L. Sequence–structure alignment was performed using Dali [85] and the secondary structure is indicated by the colored bars. The extent of the Bcl-2 homology (BH) motifs and transmembrane region (TM) is indicated by bars above the sequence and the helices below the sequences. (**b**) Table of sequence identities and similarities for the sequences in (**a**) given as percentage sequence identity/sequence similarity in each entry. Notably, the viral Bcl-2 proteins have little recognizable shared sequence identity with mammalian Bcl-2 proteins. (**c**) Profiles of BH and TM regions from Bax sequences representing bilaterians (*Lepisosteus oculatis, Strongylocentrotus purpuratus*, *Ciona intestinalis*), cnidarians (*H. vulgaris*, *Acropora digitifera*), placozoa (*T. adhaerens*), and porifera (*A queenslandica*). The height of each stack represents the conservation and the residue frequencies are represented by their height as determined by the program Skylign [86]. These indicate that the BH4 motif is a relatively weak and poorly conserved motif when compared to the BH1–BH3 motifs. Uniprot sequence and PDB IDs, hsBcl-x_L_: Q07817, 1R2D; gcBHP2: Q967D2, 5TWA; ceCED-9: P41958, 1OHU; mmBax: Q07813, 5W62; Myxoma virus M11L: Q77PA8, 2JBX; Vaccinia virus N1L: P21054, 2I39.

Bcl-2 proteins (Table 1) are identified by the presence of up to four conserved linear sequence motifs or domains comprising about 20 residues and known as Bcl-2 Homology (BH) motifs (Figure 2) [7,9].

**Figure 3:**
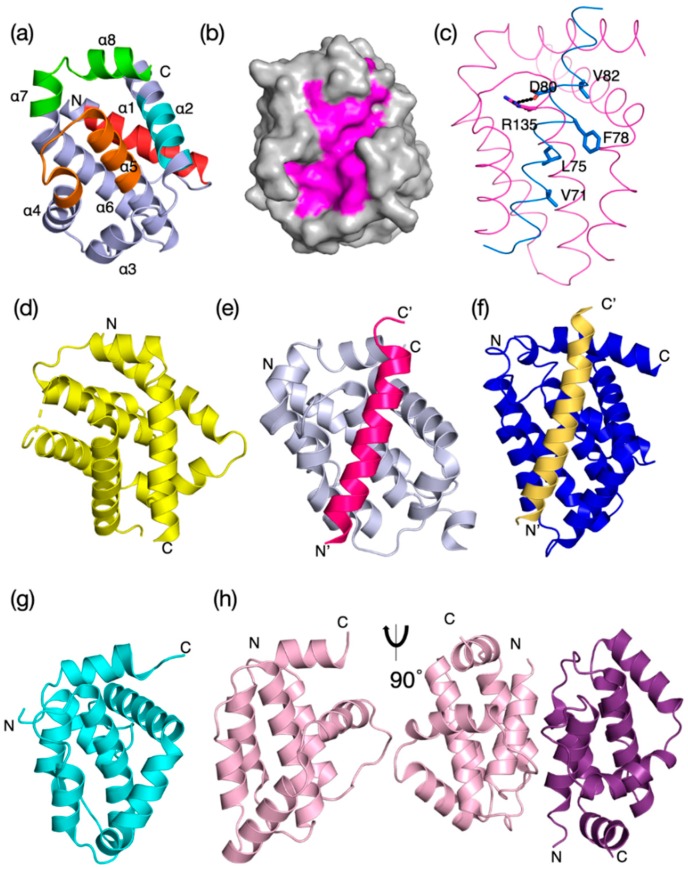
Evolutionary structure conservation in the Bcl-2 family and their complexes. Ribbon representation of the 3D structures of prosurvival and proapoptotic Bcl-2 family members and their complexes are shown. The helical bundle Bcl-2 structure occurred early in evolution and changed little over evolutionary time scales. (**a**) *H. sapiens* Bcl-x_L_ (PDB 1R2D) with BH1–4 motifs colored in orange, green, cyan, and red as shown in the sequence–structure alignment of Figure 2a, (**b**) *H. sapiens* Bcl–x_L_ (PDB 1R2D) shown as grey surface with canonical ligand-binding groove shaded in magenta, (**c**) BHP2 from the sponge *G. cydonium* Bcl-2, BHP2 (PDB 5TWA) magenta, LB–Bak sky blue. The canonical ionic interaction between the conserved Arg from prosurvival Bcl-2 and conserved Asp from the BH3 motif of prodeath Bcl-2 as well as the four conserved hydrophobic residues from the Bak BH3 motif are shown as sticks. (**d**) *M. musculus* Bax (PDB 5W62) yellow, (**e**) *H. sapiens* Bcl-b:Bim complex (PDB 4B4S), (**f**) *C. elegans* CED-9: EGL-1 complex, CED-9 (navy) with EGL–1 (sand) in the binding groove (PDB 1TY4). (**g**) Myxoma virus Bcl-2 M11L (PDB 2JBX) cyan, (**h**) Vaccinia virus N1L (PDB 2I39) salmon. Monomeric N1 is shown in the same orientation as in (**a**), and the functionally relevant dimer is shown rotated by 90^o^ around the vertical axis. In (**a**), the extent of the BH motifs is indicated as ribbon colored as in Figure 2a and the helices α1–α8 are also indicated. The structures were aligned on human Bcl-x_L_ and the orientation for all structures is the same as that in (**a**). The N and C termini are indicated.

The Bcl-2 proteins fold to form a distinct helical bundle structure where the core of the α–helical bundle is composed of a central hydrophobic helix (helix α5) that forms a scaffold for packing up to eight α-helices (Figure 2 and Figure 3). In a feature maintained from sponges to man [9], the Bcl-2 fold brings the BH regions into close proximity to assemble the canonical BH3-binding groove where an antagonist BH3 motif binds (Figure 3b,c). Whilst this “in-groove” interaction mechanism appears to be the primary mode of interaction for Bcl-2-mediated control of apoptosis, alternative modes have been proposed including a site spanning helices α1 and 6 [87] and the BH4 motif [88]. Furthermore, nonapoptotic roles including modulation of NF-κB signaling are also not mediated via an in-groove mechanism [89]. BH motifs are recognizable from the earliest metazoan Bcl-2 proteins but may be absent in viral proteins. The sequence signatures of each of the four BH motifs (BH1–BH4) differ (Figure 2c) and are found in the order from the N-terminus: BH4, BH3, BH1, BH2 (Figure 2a) and for prosurvival proteins are normally located on the same exon while the gene structure for the proapoptotic proteins is more complex. The presence of a BH3 motif is a key feature of the proapoptotic proteins and required for their proapoptotic activity [9,90], whereas some of the prosurvival proteins do not feature the BH3 motif. In addition to the presence of the BH motifs, many Bcl-2 proteins bear a C-terminal transmembrane (TM) region that is located on a separate exon. The TM region targets these proteins to intracellular membranes including the nuclear envelope, mitochondrial inner and outer membranes, Golgi apparatus, lysosomes, ER, and peroxisomes [91]. However, it is Bcl-2 family action at the mitochondrial outer membrane (MOM) that is the most central mechanistic feature for intrinsic apoptosis. Both the gene structure and synteny of Bcl-2 proteins are well conserved across phyla [92].

Our current understanding of intrinsic apoptosis is mainly derived from investigations of mouse, human, nematode (*Caenorhabditis elegans*), and fly (*Drosophila melanogaster*) apoptosis. These studies have shown the Bcl-2 family consists of two phylogenetically distinct groups of proteins: those that share the Bcl-2 fold [90,93] that are either prosurvival or proapoptotic and the proapoptotic intrinsically disordered ‘BH3-only’ proteins, that bear only the BH3 motif [9]. The BH3-only proteins are upregulated in response to diverse apoptotic stimuli [94] and their principal role is to antagonize the prosurvival proteins [9], but apoptosis also occurs in their absence [95]. Notwithstanding the conservation in the Bcl-2 family, there are substantial differences in Bcl-2-regulated apoptosis mechanisms. In mammals, there are nine multimotif Bcl-2 paralogs (six prosurvival Bcl-2, Bcl-x_L_, Bcl-w, Mcl-1, A1, Bcl-B, and three proapoptotic Bax, Bak, and Bok) and eight BH3-only proteins (Bim, Bad, Bmf, Bid, Bik, Noxa, Puma, Hrk) (Table 1) that regulate intrinsic apoptosis through a network of specific binding interactions that control the integrity of the MOM [7] (Figure 1). A key step is Bax, Bak, or Bok oligomerization at the mitochondrial membrane that results in formation of membrane pores releasing cytochrome *c* to activate the caspase cascade. In contrast to mammals, MOM permeabilization (MOMP) and cytochrome *c* do not play a role in initiation of apoptosis in ecdysozoans *C. elegans* (Figure 1) or *D. melanogaster* [7,42,96] (Figure 1). Combined, these results indicate the interactions of the Bcl-2 proteins have been maintained over the course of evolution.

The role of prosurvival Bcl-2 proteins is not limited to regulation of apoptosis; other functions have been proposed in processes as divergent as autophagy, calcium homeostasis regulation, and metabolism [97,98,99]. The most well-understood process at a molecular level of these nonapoptotic roles is that of autophagy. Both Bcl-2 and Bcl-x_L_ are able to bind the autophagy regulator Beclin-1 via a mechanism closely mimicking the canonical in-groove interaction with BH3-only proteins [49,100,101]. Beclin-1 has significant differences from the BH3-only proteins. In addition to being much longer (450 residues) than a typical BH3-only protein (54–198 residues for human BH3-only proteins), Beclin-1 has its BH3-like motif located in an unstructured N-terminal region [102] with the BH3 motif spread over the junction of two exons. In addition to its unstructured N-terminal region Beclin-1 bears a coiled coil domain and a folded evolutionary conserved domain [103]. The spread of the BH3 motif over two exons differentiates Beclin-1 from the BH3-only proteins where apart from Bid the BH3 occurs in the second-to-last exon. The molecular basis of Bcl-2 proteins in nonapoptotic functions remains to be delineated.

Dysfunctional apoptosis is one of the hallmarks of disease like cancer [104] and metazoans have coevolved with this disease [105]. While neoplasms in many vertebrates are well known [105], they have also been discovered in the early metazoans *H. vulgaris* [106], coral *Acropora palmata* [107], and molluscs [108], and in the case of hydra, occur as a result of dysregulation of apoptosis [106]. While some cancers are unique to a species, others occur across multiple species [109] and resistance to apoptosis is likely to be a central feature. There is strong interest in developing a molecular understanding in Bcl-2 function, interactions, and structures [7] but currently there is only a limited number of studies on early metazoan Bcl-2-regulated apoptosis [6,110]. However, these initial studies strongly suggest conservation of structures and mechanisms across metazoan history. Exactly how intrinsic apoptosis is manifested at a molecular level varies according to the organism, but all mechanisms rely on loss of prosurvival activity to initiate apoptosis. Consequently, there has been a drive to explore the interactions of this family and elucidate the network of functions they regulate.

## 2. Virus-Encoded Bcl-2 Homologs

The importance of the Bcl-2 family in homeostatic regulation has been exploited by viruses with many viral genomes containing a Bcl-2 protein, and in some instances, multiple Bcl-2 proteins [8]. Sequence, structural, and functional homologs of Bcl-2 are found in *Herpesviridae* as well as Nucleocytoplasmic Large DNA Viruses (NCLDVs) such as *Asfarviridae* and *Iridoviridae* [8]. Many of these virus-encoded Bcl-2 family members display substantial differences with regards to their sequence (Figure 2a,b) and interaction profiles to their mammalian proapoptotic Bcl-2 family counterparts as well as their overall structure, owing to the more rapid pace at which these proteins have evolved as part of a host–pathogen interface [8,111]. The first viral Bcl-2 homologs were identified in adenovirus [112] and the γ-herpesvirus Epstein–Barr virus (EBV) [44]. Adenoviral E1B19K was shown to be a potent inhibitor of apoptosis and could be interchanged with Bcl-2 during cellular transformation [112]. Whilst the vast majority of virus-encoded apoptosis regulatory Bcl-2 proteins act by utilizing the canonical ligand-binding groove to sequester proapoptotic Bcl-2 family members, it has become apparent that this is not the sole mechanism utilized. In addition to binding proapoptotic proteins, viruses may target host prosurvival Bcl-2 proteins through the BH3-binding groove in a manner similar to but not identical to a BH3 motif, and Hepatitis B virus X protein was shown to engage the groove of Bcl-x_L_ allowing viral replication to proceed [113].

### 2.1. Bcl-2 Homologs Encoded by Herpesviridae

Numerous *Herpesviridae* encode Bcl-2-like proteins such as BHRF1 from EBV, one of the earliest identified viral Bcl-2 homologs. BHRF1 adopts the classical Bcl-2 fold and utilizes the canonical ligand-binding groove to engage proapoptotic BH3 motif ligands [45,114]. BHRF1 was shown to prolong survival of cells [44,115], which is linked to its ability to engage proapoptotic Bcl-2 members Bim [116] and Bak [114]. An unusual herpesviral Bcl-2 homolog is found in murine γ–herpesvirus 68, M11 [117]. M11 is a potent inhibitor of TNFα and Fas-induced apoptosis, and was shown to bind multiple proapoptotic Bcl-2 proteins including Bim, Bak, and Bax [101]. However, M11 also binds the autophagy regulator Beclin-1, which bears a BH3-like motif, with nanomolar affinity (K_D_ = 40 nM), which is bound via the canonical ligand-binding groove [101]. Indeed, functional studies suggest that autophagy may be the primary cell death pathway targeted by M11 [49]. Although the majority of herpesvirus-encoded Bcl-2 proteins target intrinsic apoptosis, γ68-encoded M11 clearly shows that other cell death pathways such as autophagy can also be viable targets. Indeed, M11 is not an exception, and adenoviral E1B19K was shown to be an autophagy inhibitor via engagement of Beclin-1 [118], as was the asfarvirus African swine fever virus (ASFV) A179L (see below).

### 2.2. Poxvirus Bcl-2 Homologs

The *Poxviridae* are a large superfamily of viruses amongst the NCLDVs comprising numerous families that are characterized by their relatively large genomes (130–360 kb) that frequently encode functional and structural homologs of Bcl-2. Most notable for human disease among the pox viruses are Variola virus, the causative agent of smallpox, and Vaccinia virus, which provides the basis for smallpox vaccine. Vaccinia virus (VACV) is the prototypical member of the *Orthopoxviridae* and encodes for prosurvival F1L. VACV F1L is a potent inhibitor of intrinsic apoptosis, but displays no detectable sequence identity with mammalian Bcl-2 [119,120]. Nevertheless, structural studies revealed that VACV F1L adopts a Bcl-2 fold, albeit with a previously not observed domain-swapped topology that rendered VACV F1L a constitutive dimer [53,121]. This unusual topology is paired with a remarkably restricted ligand-binding profile, with VACV F1L only engaging Bim, Bak, and Bax. Interestingly, similar domain swapping was subsequently also observed during Bax and Bak oligomerization [122], suggesting that the structural plasticity observed amongst the virus-encoded Bcl-2 proteins is also pivotal for the function of metazoan family members. In the context of a live viral infection, F1L inhibits Bim to prevent premature host cell apoptosis [121] and replaces Mcl-1 activity [123]. An extended unstructured N-terminal section prior to the Bcl-2 fold [124] may be involved in apoptosis regulation but results are conflicting [124,125,126]. Despite being closely related to VACV F1L, the Variola virus (VAR) F1L homolog does not bind Bim; instead it binds Bid, Bak, and Bax [127], and only counters Bax-mediated apoptosis. The domain-swapped Bcl-2 topology is not restricted to the *Orthopoxviridae*, with deerpoxvirus-encoded DPV022 [55] also adopting this unusual fold [54]. Amongst the *Leoporipoxviridae*, myxomavirus encodes for antiapoptotic M11L (Figure 3d), a potent inhibitor of intrinsic apoptosis [128]. Despite lacking detectable sequence identity to cellular Bcl-2 or Bcl-x_L_, M11L adopts a Bcl-2 fold [57,129] (Figure 2a,b and Figure 3g,h) and sequesters Bax and Bak to prevent apoptosis [57], unlike VACV F1L which operates via Bim neutralization [121]. Other poxvirus-encoded vBcl-2 members include fowlpox FPV039 [58,59] and canarypox virus (CNPV058) [61], sheep poxvirus [62], and orf virus ORFV125 [63]. Outside the *herpes* and *poxviridae*, ASFV encodes A179L [130], a Bcl-2 homolog that uses the canonical ligand-binding groove to engage all major host proapoptotic Bcl-2 members [51] as well as Beclin-1 [131], thus acting as a dual apoptosis/autophagy inhibitor [50,132]. Amongst the *Iridoviridae*, grouper iridovirus (GIV) harbors prosurvival GIV66 [65] that only binds Bim [64], and forms a novel noncovalent dimeric Bcl-2 architecture which leads to an occluded ligand-binding groove [64] and dimer dissociation upon Bim binding.

While numerous *Poxviridae* encode Bcl-2 homologs that inhibit apoptosis, it has become apparent that another subset of poxviral Bcl-2 proteins exists that also modulate other functions. This group includes VACV N1 which, like M11L, has little shared sequence identity with mammalian Bcl-2 proteins (Figure 2b) but is a structural homolog (Figure 3g). N1 is a dual inhibitor of intrinsic apoptosis that adopts a dimeric Bcl-2 fold (Figure 3h) [67,133] where an additional interaction site enables modulation of NF-κB signaling that is regulated independently of the canonical Bcl-2 groove [89]. Other VACV-encoded NF-κB modulatory Bcl-2 proteins include A46 [68,134], A49 [70], A52, B14 [72], and K7 [75]. Despite targeting NF-κB signaling, substantial structural and mechanistic differences are evident across this group of Bcl-2 proteins. Although A52 and B14 utilize helices α1 and α6 to form a similar interface [72] as N1, the angle of orientation between the constituent monomers varies between the three proteins.

Intriguingly, while apoptosis inhibitory Bcl-2 members are found in *Herpes*, *Pox*, *Asfar*, and *Iridoviridae*, more specialist functions are not widely found. Although several herpesviruses as well as ASFV harbor Bcl-2 homologs with autophagy inhibitory activity, no poxvirus has been shown to inhibit autophagy via a Bcl-2 homolog. Conversely, the NF-κB inhibitory activity found in poxvirus-encoded Bcl-2 homologs is not found outside the *Poxviridae*. Whether or not these differences are attributable to the unique and fundamentally different life cycles and primary sites of infection remains to be established. These findings differentiate the viral Bcl-2 proteins from those in metazoans but indicate the diversity of interactions possible with the Bcl-2 fold.

## 3. The Nonmammalian Bcl-2 Family

Of the four nonbilaterian basal clades of metazoans, Porifera, Placozoa, Cnidaria, and Ctenophora, multiple orthologous and paralogous Bcl-2 family members have been discovered in the genomes of organisms from Porifera, Placozoa and Cnidaria, but have not yet been identified in ctenophores. In contrast to higher organisms and viruses, experimental evidence for the function of the Bcl-2 family in basal metazoans is relatively sparse. Recent sequence, structural, and biochemical evidence gained from poriferan [6], placozoan [78], and cnidarian [110] Bcl-2 family members are elucidating the mechanisms of apoptosis in basal metazoans. Furthermore, these results strongly suggest the molecular basis of intrinsic apoptosis determined by the structures, interactions, and intracellular localization of Bcl-2 proteins was foundational in metazoan evolution.

Sponges are currently considered the sister group to metazoans [135,136] and multiple Bcl-2 family proteins have been discovered in members of this phylum; for example, the genome of *Amphimedon queenslandica* contains seven potential Bcl-2 proteins [137], though little is known of their function. The demosponge *Lubormirskia baicalensis* harbors putative prosurvival and proapoptotic Bcl-2 proteins LB-Bcl-2 and LB-Bak-2 [76], and two Bcl-2 proteins, BHP1 and BHP2, have been identified in the sponge *Geodium cydonium* [77]. Structural and biochemical studies on BHP2 showed that a BH3 peptide derived from the BH3 region of *L. baicalensis* Bak-2 bound in the groove of BHP2 and many of the molecular features elucidated in mammalian Bcl-2 interactions were maintained [6]. Though the topology of BHP2 closely resembles those of other Bcl-2 proteins (Figure 2 and Figure 3), a structure–phylogenetic analysis showed there were relatively subtle differences suggesting BHP2 has unique binding features when compared to mammalian and viral Bcl-2 proteins [6]. These findings not only indicate the structure conservation but the evolutionary conservation of the intermolecular interactions of the BH3 motif:Bcl-2-in-groove interaction between prosurvival and proapoptotic Bcl-2 proteins from sponges to man.

The placozoan *Trichoplax adhaerens* has four putative Bcl-2 fold proteins in its genome [138] including two putative proapoptotic proteins, Bax (trBcl-2L3 or trBax) and Bak (trBcl-2L4 or trBak), and two prosurvival proteins, trBcl-2L1 and trBcl-2L2 [78]. TrBax is inhibited by human Bcl-2, suggesting the BH3-in-groove interaction is conserved. The putative role of trBak in *T. adhaerens* is somewhat different from that in humans, where it antagonizes the prosurvival activity of trBcl-2L1 and trBcl-2L2 rather than inducing cytochrome *c* loss from mitochondria, and thus it has been hypothesized that trBak effectively adopts the role of a BH3-only protein in mammals; however, further investigation is required to establish this proposal as no detailed interaction studies were undertaken. As for the case of *G. cydonium*, the underlying conservation of the Bcl-2:Bax interaction was demonstrated by the inhibition of trBax by human prosurvival proteins.

The BH3-only proteins play a key role in mammalian apoptosis where they antagonize the action of prosurvival proteins (Figure 1), but their presence has not been detected in the genomes of Porifera or Placozoa. However, candidates for BH3-only proteins have been detected in the cnidarian *H. vulgaris* [139,140] in addition to Bcl-2 fold sharing prosurvival proapoptotic Bcl-2 proteins. Potential BH3-only proteins have been identified in *H. vulgaris* using a yeast two-hybrid screen [110]. The relatively short sequence of the BH3-only motif with essentially only a highly conserved Leu and absolutely conserved Asp four residues downstream has made it difficult to identify bona fide BH3-only sequences by sequence alone, making it necessary for biochemical verification [9,141]. In addition to the four proposed BH3-only proteins in *H. vulgaris*, there are nine putative Bcl-2 family members including two Bak-like and seven Bcl-2 like sequences [110]. Although further studies are required to establish the exact functional relationships for these proteins, the findings of Lasi et al. [110] point to a complex signaling network for the Bcl-2 proteins even in the earliest of metazoans.

The genetic [96] and molecular and structural [79,142] foundations of Bcl-2-regulated apoptosis were established in the ecdysozoan *C. elegans* (Figure 1, Figure 2a and Figure 3f). Since these discoveries, the basis of prosurvival, proapoptotic, and BH3-only protein interaction has been verified in other organisms. The genomes of the lophotrochozoans *Schmidtea mediterranea*, *S. japonicum,* and *S. mansoni* bear multiple Bcl-2-like proteins including BH3-only components [143,144]. Investigation of apoptosis in platyhelminths (*S. mediterranea* and *Dugesia dorotocephala*) identified Bak and Bcl-2 orthologs and experimental data mitochondrial cytochrome *c* release is associated with MOMP and caspase activation [144]. Binding between the Bcl-2 proteins and BH3-only proteins in *S. japonicum* was established using immunoprecipitation experiments [143]. Mutational, structural, and biochemical studies defined the binding mode as similar to other Bcl-2:BH3 interactions and cytochrome *c* release on treatment with a BH3 motif peptide [143]. Combined, these studies on lophotrochozoans indicate that, in contrast to ecdysozoans, a tripartite mechanism exists with prosurvival, proapoptotic, and BH3-only proteins triggering MOMP and cytochrome *c* release to initiate intrinsic apoptosis in these organisms. The conclusion from these studies is that intrinsic apoptosis signaling in the protostomes has been modified by gene loss in some organisms but the underlying tripartite mechanism leading to MOMP is preserved in others and the MOM interaction remains central.

Experiments on the cytosolic extracts from the echinoderms *Strongylocentrotus purpuratus* (purple sea urchin) and *Dendraster excentricus* (sand dollar) indicate caspase activation could be induced with cytochrome *c*, suggesting mitochondrial-regulated apoptosis occurs in the deuterostomes in a similar way to that in the protostomes [144]. However, others have suggested that apoptosis activation in echinoderms may not involve cytochrome *c* release from mitochondria as cytochrome *c* is not apparently necessary for Apaf-1-activated apoptosis in the starfish *Asterina pectinifera* [145]. In the nonmammalian vertebrates, the molecular basis of apoptosis is probably best defined in zebrafish, *Danio rerio*, where all three groups of the tripartite Bcl-2 family have been identified [146]. *D. rerio* has an extensive network of Bcl-2 proteins, but as yet there are relatively few details on the mechanism of action even in this well-studied model organism [147]. Genome duplication events in teleost fish [148] have given rise to many Bcl-2 paralogs in *D. rerio* [97], but the molecular basis of apoptosis is likely similar to that in mammals [25]. Structural studies on *D. rerio* NRZ show the structure and mode of BH3 interaction is near identical to other organisms [25]. These studies establish Bcl-2 signaling in deuterostomes share many aspects with those from the protostomes and establish the basis for intrinsic apoptosis in the bilaterians.

## 4. The Role of Mitochondrial Membrane Interactions

The defining event of intrinsic apoptosis in mammals is release of cytochrome *c* from the mitochondrial intermembrane space through supramolecular pores formed by Bax, Bak, or Bok oligomerization on the MOM [149] (Figure 1). Crucial to this action is the presence of a TM anchor and most members of the Bcl-2 family bear tail anchors, including BH3-only proteins, [150] necessary both for their localization at the MOM [5] and apoptotic activity [151]. TM anchor deletion mutants of Bax and Bak lose their apoptotic abilities [152], suggesting the MOM as an activator of Bax/Bak [153]. Similarly, deletion of the TM region of prosurvival Bcl-x_L_ decreases its prosurvival activity [151]. The most striking feature of the TM regions is their poor sequence conservation [154]. Figure 2c illustrates the relatively weak conservation of the TM region compared to the BH motifs in Bax sequences. In mammalian apoptosis, cofactors such as the β-barrel Voltage Dependent Anion Channel-2 (VDAC2) may be important in Bax/Bak membrane recruitment [155], but this has not yet been demonstrated in basal metazoan apoptosis. While most investigations have focused on apoptosis in the mouse or humans, MOM association has also been observed for Bcl-2 proteins from placozoans, hydra, and viral proteins, indicating the fundamental nature of this activity to Bcl-2 action.

The structures of the proapoptotic proteins Bax, [29] Bak, [27] and Bok [156,157] have an essentially identical core that suggests a common mode of action; however, their subcellular localization and dynamics differ significantly [91]. The crystal structure of mouse Bax shows that the TM region is helical and packed in the equivalent site as occupied by EGL-1-binding CED-9 (Figure 3d,f). Prior to apoptotic stimuli, Bax is largely cytosolic [158], with a fraction being shuttled to the mitochondrion surface [159], but subsequent to apoptotic stimuli, Bax accumulates at the MOM [160,161] via a process that is dependent on its TM residues [162]. In contrast to Bax, Bak is constitutively membrane-bound and Bok is only fractionally colocalized with mitochondria [163]. The prosurvival protein Bcl-x_L_ translocates Bax from the MOM to the cytosol, and this process is dependent on the BH3-binding groove [159] and TM region of Bcl-x_L_ [164]. Thus, the proapoptotic proteins have complex membrane interactions and dynamics and emerging data supports a similar view in basal metazoans.

Experimental details on the localization and dynamics of Bcl-2 proteins are now emerging for the placozoan *T. adhaerens,* and a similar picture of complex dynamic behavior to mammalian Bcl-2 family proteins is emerging [78]. *T. adhaerens* Bcl-2 proteins are differentially partitioned between mitochondria, ER, and the cytosol, and the TM region is necessary for its membrane localization [78]. TrBcl2L1 is tightly associated with the MOM, while trBcl-2L2 (trMcl-1) and trBcl-2L4 (trBak) are cytosolic and only loosely associated with intracellular membranes [78] in a manner that mirrors the mammalian Bcl-2 proteins. Mammalian prosurvival proteins like the proapoptotic proteins are differentially partitioned between cytosol and membranes. For example, Bcl-2 is membrane integrated and Bcl-x_L_ partitioned between cytosol and membranes. Like its mammalian counterpart, Trichoplax Bax (Bcl-2L3) translocates to the mitochondria and induces cytochrome *c* release [78]. However, one caveat of these discoveries is that they have been performed in heterologous systems using expression of Trichoplax proteins in mammalian cells and have yet to be confirmed in homologous systems. The studies on *T. adhaerens* Bcl-2 proteins and their membrane interactions point to the fundamental nature of membrane interactions in their mechanism of action.

Although not all viral Bcl-2 proteins bear an obvious TM region, many do [165], and membrane interactions are necessary for their prosurvival activity. This trend closely mirrors what has been observed for mammalian proteins like Bcl-x_L_. Similarly for the viral Bcl-2 protein F1L, association with mitochondria is necessary for its prosurvival behavior [52], and M11L localizes at the mitochondria [128] and colocalizes with Bak at the MOM [166]. ASFV A179L localizes to mitochondria and ER [132]. Although C-terminal anchoring of Bcl-2 proteins is often maintained, there are exceptions; for example, vMIA targets the MOM through its N-terminal region [167]. It appears likely that the interaction of Bcl-2 proteins with membranes is fundamental to Bcl-2 function and has been conserved from the earliest metazoans and maintained in viral Bcl-2 proteins.

While MOM localization of Bcl-2 proteins may be conserved, MOMP is not necessarily maintained as a mechanism of activating apoptosis. Ecdysozoans have undergone extensive gene loss [168] and have fewer Bcl-2 genes and mechanistic differences from mammalian apoptosis (Figure 1) [169]. For example, the sole Bcl-2 protein CED-9 in the nematode *C. elegans* is TM-anchored to mitochondria like its mammalian counterpart Bcl-2 and although they have closely related structures (Figure 2 and Figure 3) and bind BH3-only proteins in their respective binding grooves, their role in apoptosis mechanisms is not identical [7]. CED-9 binds and antagonizes the apical caspase CED-4 and once released from its inhibition by the translationally upregulated BH3-only protein EGL-1 binding CED-9, CED-4 activates the caspase CED-3 [7,96]. In comparison, Bcl-2 is also localized to the MOM amongst other intracellular membranes [91] and binds BH3-only proteins but it does not bind Apaf-1, the mammalian caspase-activating protein corresponding to CED-4. Distinct from the nematode, the role of Bcl-2 proteins in the fly *D. melanogaster* is less well understood [170]. BH3-only proteins have not been found in the genome of *D. melanogaster* but two Bcl-2 proteins, Debcl and Buffy, have been recognized and it has been shown they interact [42] and both localize to the MOM with Buffy additionally found at the ER [171]. Thus, although the sequences, structure, and membrane binding may all be conserved elements in the Bcl-2 family, there are key differences in how they are manifested in the activation of caspases.

## 5. Conclusions

**Figure 4:**
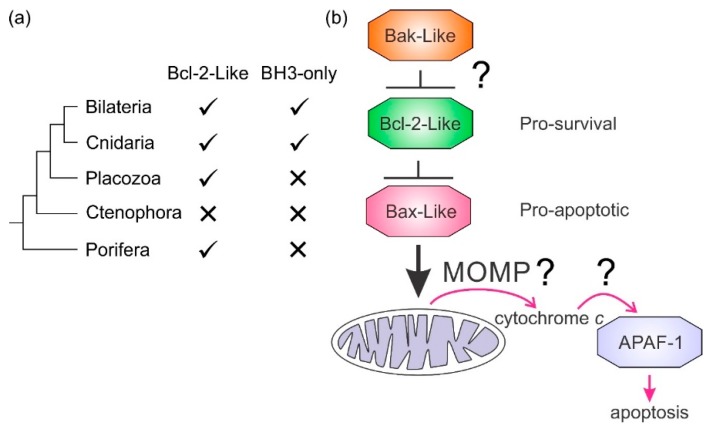
Bcl-2-like proteins in basal metazoan clades and potential apoptosis model. (**a**) Metazoan phylogenetic relationships and the presence or absence of Bcl-2 proteins. The presence or absence of Bcl-2 family members is indicated. (**b**) A simple model for intrinsic apoptosis in Porifera and Placozoa where BH3-only proteins have not been identified. Experiments have yet to delineate the roles of MOMP and adaptor protein initiation of caspases in their entirety.

It is not clear how the Bcl-2 family arose; one hypothesis is that it occurred through horizontal gene transfer from a symbiont [172], but multiple Bcl-2 genes occurred early in metazoan evolution. Even in the sponges, a phylum considered to be the sister group of all metazoans [135], multiple Bcl-2 fold proteins have been identified in genomes, such as that of *A. queenslandica* [137] where seven such proteins were recognized. In contrast to the early appearance of Bcl-2 fold proteins, BH3-only proteins have not yet been identified in Porifera or Placozoa. Ctenophores appear to have lost the genes required for Bcl-2-regulated apoptosis altogether (Figure 4a). Emerging results from biophysical and biochemical measurements performed on the nonmammalian Bcl-2 family including those from sponges [6], placozoans [78] and cnidarians [110] indicate that the basic architecture of intrinsic apoptosis is maintained for these basal metazoans. Structural studies have shown that the molecular details of interactions have been conserved from sponges to man [6] and viruses have assimilated Bcl-2 proteins [111]. A key difference between sponges, placozoans, and cnidarians is an apparent absence of BH3-only proteins in sponges and placozoans. The essential role of the BH3-only proteins, at least in mammals, is to neutralize the prosurvival proteins to allow the MOM to activate the proapoptotic Bcl-2 proteins [153]. Based on these results, a hypothesis for a simple model for intrinsic apoptosis in the absence of BH3-ony proteins could be envisaged as the prosurvival proteins keeping the proapoptotic proteins in check. Alternately, as recently proposed, the Bak-like protein may partially fulfill the role of BH3-only proteins [78] (Figure 4b). The investigation of the more evolutionary distant members of the Bcl-2 family has exposed the substantial complexity in Bcl-2-mediated signaling at the foundation of metazoan evolution and underscores the pivotal role these proteins play in biology. Functional and mechanistic studies to date have only just begun to unravel the role Bcl-2 has played during the early stages of metazoan life, and future studies are likely to discover new twists to Bcl-2 signaling.

## Figures and Tables

**Table 1:** Summary of Bcl-2-related proteins and their activities.

Name(s)	Species	Functions	References
Bcl-2	*H. sapiens*	Prosurvival	[10,11]
Bcl-w	*H. sapiens*	Prosurvival	[12,13,14]
Bcl-x_L_	*H. sapiens*	Prosurvival	[15,16]
Mcl-1	*H. sapiens*	Prosurvival	[17,18]
Bfl-1/A1	*H. sapiens*	Prosurvival	[19,20]
Bcl-b	*H. sapiens*	Prosurvival	[21,22]
Boo/Diva	*M. musculus*	Prosurvival	[21,23]
NRZ	*D. rerio*	Prosurvival	[24,25]
Bak	*H. sapiens*	Proapoptotic	[26,27]
Bax	*H. sapiens*	Proapoptotic	[28,29]
Bok	*H. sapiens*	Proapoptotic	[30]
Bad	*H. sapiens*	Proapoptotic	[31]
Bid	*H. sapiens*	Proapoptotic	[32]
Bik	*H. sapiens*	Proapoptotic	[33]
Bim	*H. sapiens*	Proapoptotic	[34]
Bmf	*H. sapiens*	Proapoptotic	[35]
Hrk	*H. sapiens*	Proapoptotic	[36]
Noxa	*H. sapiens*	Proapoptotic	[37]
Puma	*H. sapiens*	Proapoptotic	[38]
Beclin	*H. sapiens*	Proautophagic	[39]
Bcl-wav	*D. rerio*	Proapoptotic	[40,41]
Buffy	*D. melanogaster*	Proapoptotic	[42]
DeBcl	*D. melanogaster*	Prosurvival	[43]
BHRF1	Epstein–Barr virus	Prosurvival	[44,45]
KsBcl-2	Kaposi Sarcoma herpesvirus	Prosurvival	[46]
E1B19K	Human adenovirus	Prosurvival	[47]
M11	mγ68 herpesvirus	Prosurvival	[48,49]
A179L	African swine fever virus	Prosurvival	[50,51]
F1L	Vaccina virus, variola virus	Prosurvival	[52,53]
DPV022	Deer poxvirus	Prosurvival	[54,55]
M11L	Myxomavirus	Prosurvival	[56,57]
FPV039	Fowl poxvirus	Prosurvival	[58,59]
CNP058	Canary poxvirus	Prosurvival	[60,61]
SPPV14	Sheep poxvirus	Prosurvival	[62]
ORFV125	Orf virus	Prosurvival	[63]
GIV66	Grouper iridovirus	Prosurvival	[64,65]
N1	Vaccinia virus	Prosurvival, NF-κb	[66,67]
A46	Vaccinia virus	NF-κb	[68,69]
A49	Vaccinia virus	NF-κb	[70,71]
A52	Vaccinia virus	NF-κb	[72]
B14	Vaccinia virus	NF-κb	[72,73]
K7	Vaccinia virus	NF-κb, IFN signaling	[74,75]
LB-Bcl-2	*L. baicalensis*	Prosurvival	[76]
LB-Bak-2	*L. baicalensis*	Proapoptotic	[6,76]
BHP1	*G. cydonium*	Prosurvival	[77]
BHP2	*G. cydonium*	Prosurvival	[6,77]
trBcl-2L1	*T. adherens*	Prosurvival	[78]
trBcl-2L2	*T. adherens*	Prosurvival	[78]
trBcl-2L3/trBax	*T. adherens*	Proapoptotic	[78]
trBcl-2L4/trBak	*T. adherens*	Proapoptotic	[78]
EGL-1	*C. elegans*	Proapoptotic	[79,80]
CED-9	*C. elegans*	Prosurvival	[81,82]
vMIA	Cytomegalovirus	Prosurvival	[83,84]
